# Longitudinal immunomonitoring following Tocilizumab in rheumatoid arthritis

**DOI:** 10.1186/1479-5876-9-S2-P19

**Published:** 2011-11-23

**Authors:** Yves-Marie Pers, Pascale Louis-Plence, Pierre Portales, Julie Quentin, Sylvie Fabre, Jean-François Eliaou, Christian Jorgensen

**Affiliations:** 1 Inserm U844, CHU Saint-Eloi, Université Montpellier 1, Montpellier, France; 2 Unité Clinique d’Immuno-Rhumatologie et Thérapeutique Ostéo-Articulaire, CHU Lapeyronie, Montpellier, France; 3 Laboratoire d’Immunologie, Hôpital Saint-Eloi, Montpellier, France

## Introduction

Tocilizumab is a humanized anti-IL-6 receptor monoclonal antibody, which binds to circulating soluble IL-6 receptor and membrane-expressed IL-6 receptor, inhibiting IL-6 binding to both forms of IL-6 receptor. Tocilizumab is an efficient therapy for adults with moderate to severe RA in whom DMARDs or a TNF inhibitor has failed. However, the impact of tocilizumab on several immune populations such as lymphocytes, monocytes, dendritic cells is still unknown. In order to longitudinally analyze the variations in frequencies as well as the activation status of these populations we designed several panels specific for immune cells and performed immunomonitoring by FACS analyses.

## Patients and methods

20 patients with severe and active RA, refractory to methotrexate or anti-TNF therapies were recruited and treated with 8mg/kg of tocilizumab monthly. Peripheral blood was recovered for each patient at day 0, as well as 1 and 3 month after informed consents. Staining was performed on whole blood and Peripheral Blood Mononuclear Cells were purified for intracellular cytokine staining following CD3-CD28 overnight stimulation. The data were acquired on a FACS Canto II (8 colors) and analyzed using DIVA software. 10 panels specific for sub-populations of T cells (Th1, Th2, Th17), B cells (naïve, memory and transitional), monocytes (classical, inflammatory and non-classical) dendritic cells (myeloid and plasmacytoid), Tregs (natural and induced) were designed. The activation status of the T, B and DC was also monitored.

## Results

According to the cytokinic secretion of the T cells following ex-vivo activation, we observed various profile of patients: Th1, Th17 or Th1/Th17. The frequencies of these pro-inflammatory cytokine secreting T cells were found to be decreased following treatment with tocilizumab. Concerning the longitudinal analyses of the B cell populations, an increase of the transitional B cells (CD24^high^CD38^high^) was observed in non responder patients and in contrast an increase of non classical monocytes was observed in good responders. Concerning the Treg population, the high heterogeneity of the patients before treatment was confirmed. However the CD4CD25FoxP3 staining was confined to CD127^low^ and CD62L^high^ suggesting the induction of induced Tregs following Tocilizumab treatment as observed after infliximab treatment.

## Conclusion

The design of specific panels to analyse the frequencies and the activation status of several sub-population of immune cells, allows a longitudinal monitoring of the major effector populations in RA patients following cytokine blockade.

